# A co-infection of varicella-zoster virus and *Pneumocystis jirovecii* in a non-HIV immunocompromised patient: a case report

**DOI:** 10.1186/s12879-019-4715-7

**Published:** 2019-12-30

**Authors:** Hirotada Muramatsu, Akira Kuriyama, Yoshiaki Anzai, Tetsunori Ikegami

**Affiliations:** 1 Emergency and Critical Care Center, Kurashiki Central Hospital, 1-1-1 Miwa, Kurashiki Okayama, 710-8602 Japan; 2 Department of General Medicine, Kurashiki Central Hospital, 1-1-1 Miwa, Kurashiki Okayama, 710-8602 Japan

**Keywords:** Coinfection, Immunocompromised hosts, Opportunistic infections, *Pneumocystis jirovecii*, Varicella-zoster virus

## Abstract

**Background:**

Varicella-zoster virus (VZV) causes herpes zoster. *Pneumocystis jirovecii* (PJ) also causes pneumonia in immunocompromised hosts. Although both cause opportunistic infections, it is rare to have a co-infection in a non-human immunodeficiency virus carrier.

**Case presentation:**

An 84-year-old woman with hemolytic anemia referred because of acute respiratory failure. She had received prednisolone without PJ pneumonia prevention. She developed dyspnea and desaturation while eating, and thus was treated based on a presumptive diagnosis of aspiration pneumonia. Physical examination revealed a vesicular rash on the left side of her neck suggesting herpes zoster infection. Polymerase chain reaction of her sputum for PJ and VZV was positive, which confirmed a diagnosis of pneumonia due to PJ and VZV co-infection. Despite acyclovir and sulfamethoxazole and trimethoprim administration, she died on hospital day 19.

**Conclusions:**

Clinicians should suspect PJP when patients on systemic corticosteroids develop pneumonia and they have not received prophylactic treatment for PJP in non-HIV carriers. When such patients have a VZV rash, clinicians should aggressively seek signs of opportunistic infections. Our case hereby highlights the importance of recognizing the possibility of a VZV and PJ co-infection.

## Background

Varicella-zoster virus (VZV) rarely causes pneumonia in immunocompromised or immunocompetent hosts. *Pneumocystis jirovecii* (PJ) causes life-threatening pneumonia, known as *Pneumocystis jirovecii* pneumonia (PJP), in immunocompromised hosts. Both pathogens cause known opportunistic infections, but co-infection with both entities in a non-human-immunodeficiency-virus (HIV) carrier is uncommon. We describe a case of VZV and PJ co-infection in a non-HIV carrier on chronic systemic corticosteroids.

## Case presentation

**Fig. 1:**
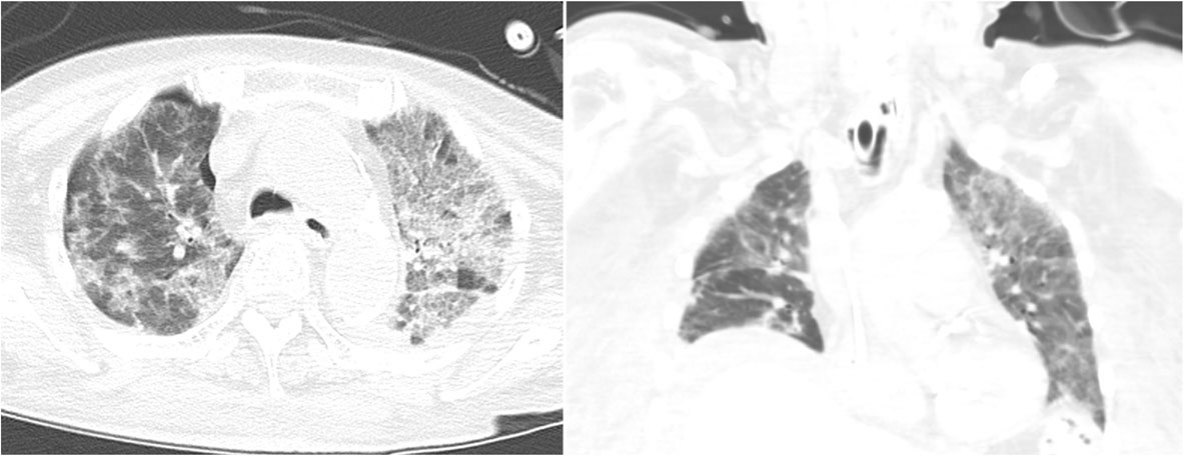
New consolidation with ground glass opacity in the patient’s lungs

An 84-year-old woman with a recent diagnosis of hemolytic anemia referred to our hospital because of acute respiratory failure. During the 64 days before this referral, the patient received prednisolone titrated between 10 mg and 80 mg daily. As of the referral, she received 40 mg/day of prednisolone. A few days prior, she developed a fever without any accompanying symptoms. On the day of the referral, she developed dyspnea, desaturation, and altered mental status while eating, and was transported to our hospital. We supposed that the patient inspired food while eating and that inspiration-induced hypoxia caused deterioration of her mental status. Her vital signs on arrival were a body temperature of 37.0 °C, heart rate of 150 beats/min, blood pressure of 170/70 mmHg, respiratory rate of 24/min, and 88% oxygen saturation on a reservoir mask of 10 L/min. Her Glasgow Coma Scale (GCS) was 7 (E2V1M4). She had hypoxemia refractory to full mask oxygenation and was eventually intubated in the emergency room. The white blood cell (WBC) count was 9200 cells/mcL (neutrophils, 97.5%; eosinophils, 0.0%; basophils, 0.0%; lymphocytes, 1.5%; and monocytes, 1.0%). Computed tomography (CT) identified patchy infiltrates along the bronchi in her right lung, with consolidation and ground-glass opacity in her left lung (Fig. 1). Physical examination revealed diminished right breath sounds and a vesicular rash on the left side of her neck. We diagnosed the rash as herpes zoster based on cutaneous findings and initiated valacyclovir (1 g daily). Because her oxygenation and mental status deteriorated while eating, we suspected aspiration pneumonia and sulbactam/ampicillin (3 g every 12 h) was initiated. Prednisolone (5 mg daily) was continued because of her corticosteroid history.

Her mental status remained unchanged after oxygenation recovery following tracheal intubation. Cerebrospinal fluid analysis, brain CT and magnetic resonance imaging, and electroencephalogram were non-contributory. To exclude valacyclovir-induced encephalopathy as a cause of her coma, we discontinued valacyclovir on hospital day 6.

Sputum cultures on admission and on hospital day 4 were positive for *Klebsiella pneumoniae,* extended-spectrum-beta-lactamase-producing *Escherichia coli*, and *Corynebacterium striatum*. We changed her antibiotics to cefmetazole (2 g every 12 h) and vancomycin (500 mg to 1 g at a time). However, both respiratory status and pneumonia were refractory to the current antibiotic regimen and her condition worsened. Specifically, the worst PaO_2_/FiO_2_ ratio was 120. The β-D-glucan value measured on hospital day 6 was elevated at 285.6 pg/mL (reference, 0.0–19.9 pg/mL). We started sulfamethoxazole and trimethoprim (7.5 mg/kg/day) therapy, and increased her prednisolone to 80 mg/day to treat the presumptive PJP. Polymerase chain reaction (PCR) of her sputum for PJ and VZV was positive, which confirmed a diagnosis of pneumonia due to PJ and VZV co-infection. We added intravenous acyclovir (500 mg every 12 h). At that time, we observed eschars on her left neck and external ear canal, which suggested systemic VZV infection. The patient remained comatose and her respiratory status and renal function continued to decline despite the treatment. The patient’s family opted for palliative care and she died on hospital day 19.

## Discussion and conclusions

This was a case of PJ and VZV co-infection. Systemic corticosteroid use can suppress the immune system, thereby rending the patient susceptible to opportunistic infections.

PJ colonizes in more than 50% of immunocompetent adults [1]. In immunocompromised hosts, PJ can cause severe respiratory failure and is often fatal. PJP in human immunodeficiency virus (HIV) carriers is usually asymptomatic and slowly causes desaturation. In contrast, non-HIV carriers with PJP have rapidly progressive and severe respiratory symptoms, and a relatively high mortality rate of 30–60%.

VZV causes a characteristic vesicular rash called varicella (chickenpox) or herpes zoster (shingles). VZV is occasionally associated with serious complications such as central nervous system disorders, pneumonia, arthritis, osteomyelitis, necrotizing fasciitis, and secondary bacterial infections, including sepsis [2]. Varicella-zoster pneumonia (VZP) is a rare clinical condition; < 5% of healthy individuals develop VZP, while 5–10% of immunocompromised hosts present with herpes zoster [3, 4]. VZP is rare, even in patients with herpes zoster. Patients with VZP have the rash for a few weeks. The manifestations of VZP are non-specific and noticeable 3–5 days after the appearance of skin lesions [5]. Initial respiratory symptoms include cough, which is seen in 11% of patients with VZP. Although VZP can cause acute respiratory distress syndrome, it rarely leads to death.

There are a limited number of reports in the literature of co-infections of PJ with members of the Herpesviridae family, such as cytomegalovirus and human herpesvirus type 6, in immunocompromised HIV and non-HIV hosts (Table 1) [6–8]. Kosmidis et al. found a patient with cytomegalovirus and PJ co-infection in a prospective analysis of 58 leukemic children [6]. This child died because of severe interstitial pneumonia. Vetter et al. reported a co-infection of cytomegalovirus and PJ in a 70-year-old woman treated with methotrexate and prednisone for large-vessel vasculitis [7]. Her first symptoms were fever, nausea, and dyspnea and developed pneumonia. Methotrexate was discontinued and the dose of prednisone was reduced, and she had a complete recovery after she received intravenous pentamidine and ganciclovir. Vuorinen et al. reported a human herpes virus type 6 and PJ co-infection in a 31-year-old man with hypogammaglobulinemia [8]. He presented with fever, non-productive cough, and dyspnea and had severe interstitial pneumonia. Thus, co-infection of VZV and PJ, as seen in our patient, is possible. Ostermann et al. identified VZV co-infections in a few cases with PJP [9]. Clinicians need to recognize the potential for co-infection with these two entities and its high pathogenicity.

**Table 1:** Coinfection of *Pneumocystis jirovecii* and members of herpesviridae in immunocompromised patients

Author/ Year	Age	Sex	Underlying disease	Herpesviridae	Symptoms or diagnosis	Prognosis
Kosmidis/ 1980	unknown (child)	unknown	acute leukemia	CMV	Dx: interstitial pneumonia	death
Vuorinen/ 2004	31	male	hypogammaglobulinemia	HHV-6	Dx; interstitial pneumonia Symptoms: fever, non-productive cough, dyspnea	full recovery
Vetter/ 2010	70	female	large-vessel vasculitis	CMV	Dx: pneumonia fever, nausea, dyspnea	full recovery

Abbreviations: *Dx* diagnosis, *CMV* cytomegalovirus, *HHV-6* human herpesvirus 6

We might have missed opportunities to diagnose this patient with VZV or PJ co-infection. First, our patient used prednisolone for 2 months without administration of sulfamethoxazole/trimethoprim as preventative treatment for PJP. This rendered the patient susceptible to PJP. Second, when her pneumonia was refractory to antibiotics, we could have considered other pathogens, including PJ and VZV. Because she had herpes zoster on admission, systemic VZV infection could have been considered. In HIV carriers, CD4+ cell counts are usually measured to estimate the risk of opportunistic infections. Herpesviridae pneumonia can develop later than PJP in the course of acquired immunodeficiency syndrome [10]. In contrast, we do not routinely measure CD4+ cell counts in non-HIV carriers and we were unaware of the risk of VZV pneumonia. Therefore, we could have identified these complex conditions earlier in her treatment by simply recognizing that she was immunocompromised, had not received prophylactic treatment for PJP, and had a concurrent herpes zoster infection.

Clinicians should suspect PJP when patients on systemic corticosteroids develop pneumonia and they have not received prophylactic treatment for PJP in non-HIV carriers. When such patients have a VZV rash, clinicians should aggressively seek signs of opportunistic infections. Our case hereby highlights the importance of recognizing the possibility of a VZV and PJ co-infection.

## Data Availability

All data generated or analysed during this study are included in this published article.

## Abbreviations

CT: Computed tomography
HIV: Human Immunodeficiency Virus
PJ: *Pneumocystis jirovecii*
PJP: *Pneumocystis jirovecii* pneumonia
VZV: Varicella-zoster virus

## References

[1] Ponce CA, Gallo M, Bustamante R, Vargas SL (2010). Pneumocystis colonization is highly prevalent in the autopsied lungs of the general population. Clin Infect Dis.

[2] Heininger U, Seward JF (2006). Varicella. Lancet (London, England).

[3] Marin M, Watson TL, Chaves SS, Civen R, Watson BM, Zhang JX, Perella D, Mascola L, Seward JF (2008). Varicella among adults: data from an active surveillance project, 1995-2005. J Infect Dis.

[4] Feldman S (1994). Varicella-zoster virus pneumonitis. Chest.

[5] Chiner E, Ballester I, Betlloch I, Blanquer J, Aguar MC, Blanquer R, Fernandez-Fabrellas E, Andreu AL, Briones M, Sanz F (2010). Varicella-zoster virus pneumonia in an adult population: has mortality decreased?. Scand J Infect Dis.

[6] Kosmidis HV, Lusher JM, Shope TC, Ravindranath Y, Dajani AS (1980). Infections in leukemic children: a prospective analysis. J Pediatr.

[7] Vetter M, Battegay M, Trendelenburg M (2010). Primary cytomegalovirus infection with accompanying Pneumocystis jiroveci pneumonia in a patient with large-vessel vasculitis. Infection.

[8] Vuorinen T, Kotilainen P, Lautenschlager I, Kujari H, Krogerus L, Oksi J (2004). Interstitial pneumonitis and coinfection of human herpesvirus 6 and Pneumocystis carinii in a patient with hypogammaglobulinemia. J Clin Microbiol.

[9] Ostermann A, Klueppelberg U, Wassermann K, Krueger GR (1994). Non-specific interstitial pneumonia (NIP): immunohistologic screening of etiologic agents. *In vivo* (Athens, Greece).

[10] Huang L, Crothers K (2009). HIV-associated opportunistic pneumonias. Respirology (Carlton, Vic).

